# Genetic Elements Associated With Antimicrobial Resistance Among Intestinal Bacteria

**DOI:** 10.5812/jjm.9924

**Published:** 2014-05-01

**Authors:** Bita Bakhshi, Nazanin Eftekhari, Mohammad Reza Pourshafie

**Affiliations:** 1 Department of Bacteriology, Faculty of Medical Sciences, Tarbiat Modares University, Tehran, IR Iran; 2 Department of Biology, Faculty of Basic Science, Science and Research Branch, Islamic Azad University, Tehran, IR Iran; 3 Department of Bacteriology, Pasteur Institute of Iran, Tehran, IR Iran

**Keywords:** Integrons, Drug Resistance, Antimicrobial Agents, Intestinal Bacteria

## Abstract

**Background:**

Integrons are the major reasons of multidrug resistance (MDR) among enteropathogenic bacteria. Occurrence of horizontal gene transfer between integron-carrying microorganisms and other enteric bacteria may increase the rate of emergence of integron-associated antibiotic resistance.

**Objectives:**

The objective of this study was to investigate class 1 integrons among members of enteropathogenic bacteria isolated from patients in Iran.

**Materials and Methods:**

A total of 120 enteropathogenic bacterial isolates from diarrhoeal patients were included in this study. Identities of the isolates were investigated by biochemical tests and confirmed by genus or species specific PCRs. Antimicrobial susceptibility testing was performed according to the Clinical and Laboratory Standards Institute (CLSI) guidelines. Presence of class 1 integron among the isolates was investigated using primers specific for the integrase gene conserved region.

**Results:**

The result of this study showed the highest resistance to trimethoprim and cotrimoxazole, especially in enteropathogenic *Escherichia coli* (EPEC) (100%), *Shigella sonnei* (93.7%) and *Vibrio cholerae* (95%). The results showed that 16 (57.1%) of the 28 EPEC, 9 (25%) of the 36 *Salmonella enterica*, 32 of the 13 (40.6%) *Sh. sonnei*, and only 1 (4.2%) of the 24 *V. cholerae* isolate harbored class 1 integron.

**Conclusions:**

The data obtained in the present study suggested that class 1 integrons are widely distributed among members of *Enterobacteriaceae*. The resistance patterns of our *E. coli*, *S. sonnei*, and *S. enterica* isolates were nearly identical, suggesting the same genetic elements involved in attainment of multi-drug resistance.

## 1. Background

Diarrhea occurs worldwide and is most commonly caused by enteropathogenic bacterial infections, killing annually around 2.2 million people globally, mostly children, in developing countries (1). Because of the increased resistance to most of the widely-used antibiotics, effective treatment has become more difficult in recent years. It has been shown that the resistance genes present on the plasmids and transposons of Gram-negative bacteria can be integrated into DNA elements called “integrons” (2). Integrons are the major reason of multidrug resistance (MDR) in enteropathogens (3). 

Occurrence of horizontal gene transfer between integron-carrying microorganisms and other enteric bacteria may increase the emergence rate of integron-associated antibiotic resistance (4, 5). As the case for most other antibiotic-resistant pathogens, the spread of integron-carrying MDR enteropathogens has usually been detected during outbreaks of nosocomial infection (4, 6). Integrons can carry a variety of antibiotic resistance genes, inserted as cassettes (7). There are five different integron classes with different integrated gene cassettes, among which class 1 and 2 integrons are most commonly found within enteropathogens (8). 

Integrons are common in nature, and class 1 integrase gene, intI1, is detected as the majority of integrons found in clinical isolates (7). This integron class is the most widely studied and defined. Integrons have been found among clinical and commensal isolates of both humans and animals. Even though more than 60 drug-resistance genes have been identified in class 1 integrons, it is becoming apparent that the common gene cassettes are those associated with aminoglycoside and trimethoprim resistance. Moreover, studies have clearly demonstrated swapping of these antibiotic resistance genes into integrons; therefore, presenting integrons as important tools in the rapid evolution and dissemination of antibiotic re sistance among Gram-negative microorganisms (9).

## 2. Objectives

The aim of this study was to identify class 1 integrons among members of enteropathogenic bacteria, including *Salmonella enterica*, *Shigella sonnei*, enteropathogenic *E. coli* (EPEC) and *Vibrio cholerae* strains, isolated from patients in Iran.

## 3. Materials and Methods

### 3.1. Clinical Isolates

A total of 120 enteropathogenic bacterial isolates from stool samples of diarrhoeal patients were included in this study (Table 1). The isolates were randomly selected from our bacterial collection, gathered during a 3-year period from Tehran province. Biochemical screening tests and serogrouping were performed to characterize the *Sh. sonnei*, *S. enterica*, EPEC and *V. cholerae*, and the identities of isolates were confirmed using primer sets, which specifically detected these enteropathogens as described elsewhere (Table 1).

**Table 1.** Primers Used in This Study

Target	Primer Sequences 5'-3'	Amplicon Size, bp	Reference
**Enteropathogenic ** ***E. coli*** ** (eae)**		422	(10)
	ACACTCCGATTCCTCTGGTG		
	CTTGCACATAAGCAGGCAAA		
***Salmonella enterica*** ** (invA)**		243	(11)
	ACAGTGCTCGTTTACGACCTGAAT		
	AGACGACTGGTACTGATCGATAAT		
***Shigella*** ** spp.(ipaH)**		620	(12)
	GTTCCTTGACCGCCTTTCCGTTACCGTC		
	GCCGGTCAGCCACCCTCTGAGAGTAC		
***Vibrio cholera*** ** (16S-23S intergenic region)**		300	(13)
	AGTCACTTAACCATTCAACCCG		
	TTAAGCGTTTTCGCTGAGAATG		
**Class 1 integron (int1)**		900	(14)
	TGCGTGTAAATCATCGTCGT		
	CAAGGTTCTGGACAGTTGC		

### 3.2. Antimicrobial Susceptibility Testing

The antimicrobial agents were selected among those commonly carried by integrons. Antimicrobial susceptibility testing was performed using disc diffusion method according to the Clinical and Laboratory Standards Institute (CLSI) guidelines for each bacterial species (15, 16), for ampicillin (10 µg), chloramphenicol (30 µg), streptomycin (10 µg) (Becton Dickinson, Sparks, Maryland, USA), cotrimoxazole (25 µg), tetracycline (30 µg), ciprofloxacin (5 µg) (Difco Laboratories, Detroit, Michigan, USA), and trimethoprim (Mast Diagnostics Ltd, Bootle, Merseyside, UK) (5 µg) (15, 16). The antimicrobials were classified as aminoglycosides (STR, AMP), tetracyclines (TE), amphenicols (CHL), fluoroquinolones (CIP), trimethoprim alone (TMP), or in combination with sulfonamides (SXT).

### 3.3. Integron Characterization and Sequencing of Resistance Gene Cassettes

The total DNA was extracted by boiling method and used as template in the PCR assays for pathogen confirmation and class1 integrons detection. The primer sequences used in this study are shown in Table 1. PCR was performed in a reaction mixture with total volume of 25 µL, containing 2.5 µL 10x Taq polymerase buffer, 0.3 µL dNTPs (10 mmol/L), 1 U Taq DNA polymerase, 0.6 µL MgCl_2_ (50 mmol/L), and 0.3 mol/L of each primer. PCR was performed as follows: initial denaturation step at 94°C for 5 minutes, followed by 30 cycles consisting of denaturation (94°C for 1 minute), annealing (56°C for 1 minute, separately set for each primer pair), and extension (72°C for 1 minute), followed by a final extension step at 72°C for 3 minutes. One representative of each amplified fragment (eae, ipaH, invA, prVC) was purified using QIAquick gel extraction kit (Qiagen) and subjected to direct sequencing using ABI 3730X capillary sequencer (Genfanavaran, Macrogen, Seoul, Korea). Further analysis of the se quenced fragments was performed using BLAST software.

## 4. Results

### 4.1. Clinical Isolates

A total of 120 enteropathogenic bacteria were included in this study. Twenty eight EPEC, 36 *S. enterica*, 32 *Sh. Sonnei,* and 24 *V. cholerae* were randomly selected from our collection, and conventional biochemical tests and PCR analysis confirmed the identity of the isolates.

### 4.2. Antimicrobial Susceptibility Analysis

The highest resistance among the total enteropathogenic bacteria was observed against trimethoprim and cotrimoxazole, especially among EPEC (100%), *Shigella* spp (93.7%) and *V. cholerae* (95%) isolates (Table 2). All EPEC were resistant to all tested antibiotics. The foremost resistance profile among *Sh. sonnei* was SXT/TE/TMP (34.5%). The most prevalent resistance was seen to trimethoprim and cotrimoxazole (93.7%) and tetracycline (87.5%), while only one isolate (3.1%) was resistant to ciprofloxacin. The most prevalent resistance pattern among *V. cholerae* isolates was SXT/TMP/STR/CHL (33.3%). 

The highest resistance rate was seen to SXT (95%), streptomycin (95%), and chloramphenicol (91.7%), and the least resistance was seen to ciprofloxacin (8.3%) and ampicillin (12.5%), while no resistance was seen to tetracycline. Most of the strains (95%) were MDR (resistant to ≥ 3 classes of antimicrobial agents), however, with very unlike combinations of antibiotics. The foremost resistance profile among *S. enterica* isolates was SXT/TMP/TE/ STR (13.8%). The most prevalent resistance was seen to streptomycin and tetracycline (47.2%), while only 1 (2.8%) isolate was resistant to chloramphenicol (Table 2).

**Table 2.** Antimicrobial Susceptibility Testing of the Isolates ^a^, ^b^

Bacteria	SXT	TE	TMP	STR	CIP	CHL	AMP
**EPEC**	28 (100)	28 (100)	28 (100)	28 (100)	28 (100)	28 (100)	28 (100)
***S. entarica***	10 (27.8)	17 (47.2)	10 (27.8)	17 (47.2)	4 (11.1)	1 (2.8)	9 (25)
***Sh. sonnei***	30 (93.7)	28 (84.3)	30 (93.7)	13 (40.6)	1 (3.1)	7 (18.7)	12 (37.5)
***V. cholerae***	23 (95.8)	0 (0)	23 (95.8)	23 (95.8)	2 (8.3)	22 (91.7)	3 (12.5)
**Total**	91 (75.8)	73 (60.8)	91 (75.8)	81 (67.5)	35 (29.2)	58 (48.3)	52 (43.3)

^a^ Abbreviations: AMP, aminoglycosides; CHL, amphenicols; CIP, fluoroquinolones; STR, ; SXT, sulfonamides; TE, tetracyclines; TMP, trimethoprim alone.

^b^ Data are presented in No. (%).

### 4.3. Polymerase Chain Reaction Analysis of Class 1 Integrons

**Figure 1.**
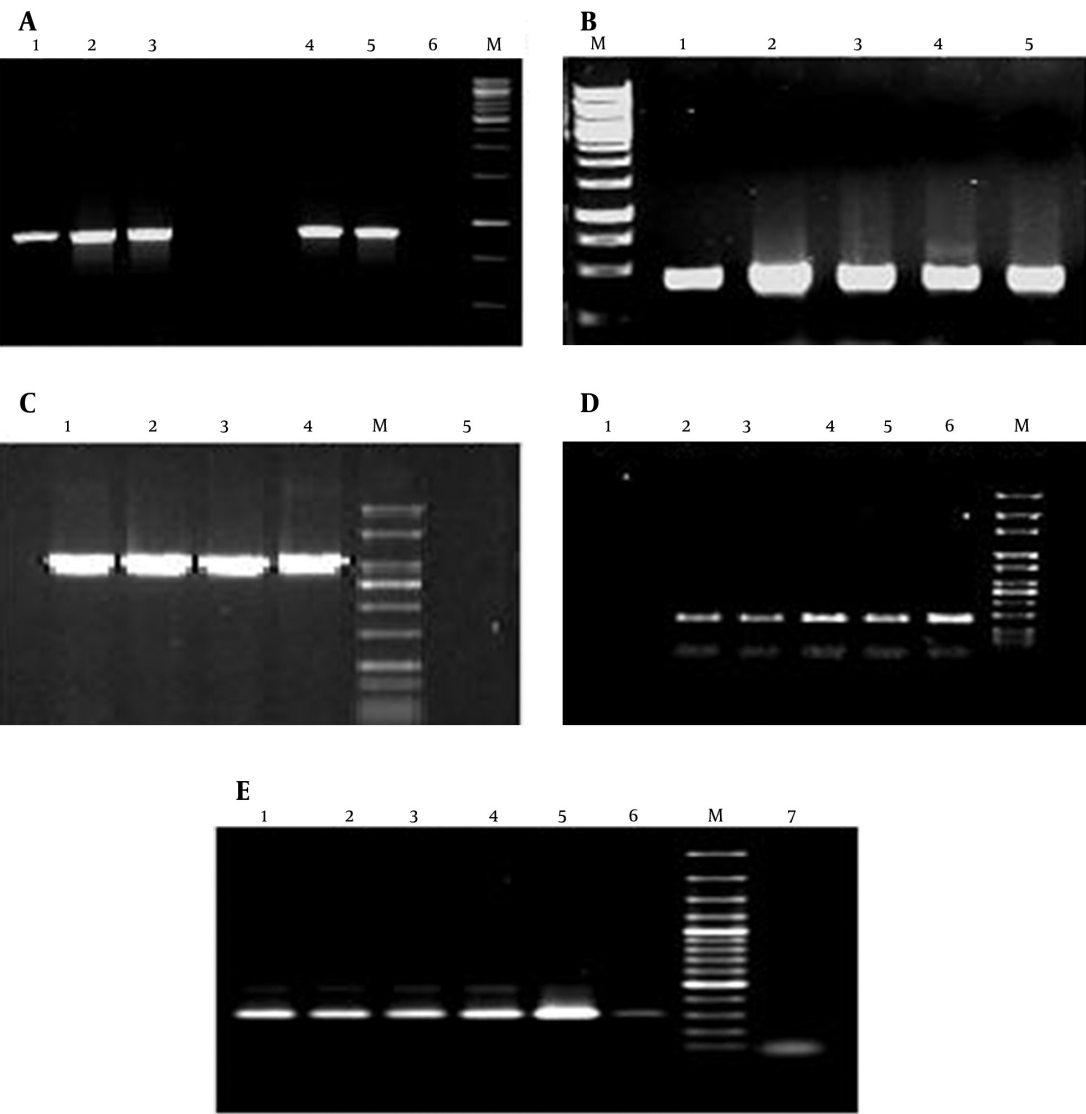
PCR Amplifications Performed in This Study. A) PCR amplification of integrase gene (int) of class 1 integron; lanes 1-4: int+ isolates, lane 5: positive control, lane 6: negative control. B) PCR amplification of eae gene; lanes 1-4: eae+ isolates, lane 5: positive control. C) PCR amplification of ipaH gene; lanes 1-3: ipaH+ isolates, lane 4: positive control, lane 5: negative control. D) PCR amplification of hilA gene; lanes 3-6: hilA+ isolates, lane 2: positive control, lane 1: negative control. E) PCR amplification of 16s-23s intergenic spacer region of *V. cholerae*; lanes 2-6: positive isolates, lane 1: positive control, lane 7: negative control.

Species- or genus-specific PCR analyses confirmed the identity of the isolates. All 120 isolates were subjected to class 1 integron (int+) analysis using primers int1-F and int1-R (5'-conserved region) with an amplification band of 900 bp (Figure 1). The results showed that 16 (57.1%) of 28 EPEC, 9 (25%) of 36 *S. enterica*, 13 (40.6%) of 32 *Sh. sonnei*, and only 1 (4.2%) of 24 *V. cholerae* isolates were positive for conserve region of class 1 integron.

## 5. Discussion

The results of this study indicated high prevalence of resistance to cotrimoxazole and trimethoprim among all the studied species. This finding was in agreement with other studies from Europe and Asia (17, 18), reporting MDR among members of enterobacterial species, with a high percentage of resistant isolates to cotrimoxazole and trimethoprim. In this study, the observed high resistance to cotrimoxazole and trimethoprim (75.8%) and streptomycin (67.5%) suggested probable dominance of the associated resistance gene cassettes within variable region of class 1 integrons, among the isolates. 

In this study, class 1 integron was identified in all species and the most prevalence was seen among EPEC isolates (57.5%), while only one isolate of *V. cholera*e harbored class 1 integron. Twenty five percent of *S. enterica* and 37.5% of *Sh. sonnei* contained class 1 integron, respectively. Recent studies have clearly illustrated the widespread distribution of class 1 integrons among bacterial isolates in Europe (19), the USA, Africa (20) and Asia (21). Emergence of MDR pathogens among *Enterobacteriaceae* is highly related to integrons, particularly class 1 integrons (22). Reyes et al. showed that class 1 integrons were common (32.4%) among 191 enterobacterial species (23). In a study by Zhu and colleagues, high prevalence of MDR (95.6%) was observed among the 90 *Shigella* strains, isolated from stool of patients in china, and 87.8% of the MDR strains carried class 1 integrons (3.3%) (17). According to a study by Ibrahim et al. the prevalence of class 1 integron was 95% in the outbreak isolates and 97% in sporadic cases (24). Ibrahim and colleagues reported occurrences of class 1 integron among 40.6% of EPEC isolated from Sudan (24). In Iran, distribution of class 1 integron in *Salmonella* isolates in Tehran was 38.1% (25). This shows an increase in the prevalence of class 1 integron among *salmonella* isolated of Iranian population.

The data obtained in the present study suggested that class 1 integrons were more widely distributed among members of *Enterobacteriaceae* than other families of enteropathogenic bacteria including vibrionaceae. In older studies, it was suggested that the integrons were significantly correlated with resistance to certain antibiotics including gentamicin, kanamycin, streptomycin, tobramycin, sulfafurazole, trimethoprim, ampicillin, chloramphenicol, and tetracycline (26). The resistance patterns present in our *E. coli*, *Sh. sonnei*, and *S. enterica* isolates were nearly identical, suggesting that probably these different species have similar mechanisms for attainment of MDR. All of these results together with earlier finding of the extremely efficient interspecies transfer of integron-carrying elements, illustrated that horizontal transfer of integron-carrying elements plays a dominant role in the development of MDR among members of *Enterobacteriaceae*, independent of species or origin of isolation (10).

The findings signified the need for monitoring antimicrobial resistance among different members of *Enterobacteriaceae*, as well as their integron distribution and content. Moreover, further studies are needed to understand the exact mechanisms involved in spreading of integrons among different bacterial genera.
